# Intuitive Face Judgments Rely on Holistic Eye Movement Pattern

**DOI:** 10.3389/fpsyg.2017.01005

**Published:** 2017-06-20

**Authors:** Laura F. Mega, Kirsten G. Volz

**Affiliations:** ^1^Werner Reichardt Centre for Integrative NeuroscienceTübingen, Germany; ^2^ University of Tübingen, *Tübingen*, Germany

**Keywords:** intuition, eye-tracking, holistic processing, intuitive judgment, face perception

## Abstract

Non-verbal signals such as facial expressions are of paramount importance for social encounters. Their perception predominantly occurs without conscious awareness and is effortlessly integrated into social interactions. In other words, face perception is intuitive. Contrary to classical intuition tasks, this work investigates intuitive processes in the realm of every-day type social judgments. Two differently instructed groups of participants judged the authenticity of emotional facial expressions, while their eye movements were recorded: an ‘intuitive group,’ instructed to rely on their “gut feeling” for the authenticity judgments, and a ‘deliberative group,’ instructed to make their judgments after careful analysis of the face. Pixel-wise statistical maps of the resulting eye movements revealed a differential viewing pattern, wherein the intuitive judgments relied on fewer, longer and more centrally located fixations. These markers have been associated with a global/holistic viewing strategy. The holistic pattern of intuitive face judgments is in line with evidence showing that intuition is related to processing the “gestalt” of an object, rather than focusing on details. Our work thereby provides further evidence that intuitive processes are characterized by holistic perception, in an understudied and real world domain of intuition research.

## Introduction

The intuitiveness of rapid perceptions of race, gender, ethnicity, and emotional state of other persons has been reliably demonstrated. In other words: “Intuition is essential to optimal social and interpersonal functioning” (Ambady, 2010). Understanding this intuitive processing of (social) information is of utter importance for general society and policy makers alike. It lies at the basis of understanding social interactions in general, as well as specific phenomena such as impression formation, person perception and adaptive social behavior. While the term intuition does surface now and again within social judgment research, the context of face perception remains understudied thus far within the intuition research community. For this reason, the present work uses the context of face perception paradigms as a means of investigating intuitive judgment processes.

Even though many of our human experiences rely on intuition, a clear scientific definition of intuition remains elusive. Intuition has often been theoretically described through the demarcation by a second “type of thinking” (Evans, 2008; Witteman et al., 2009), namely slow and effortful deliberation. This dualistic distinction is ancient in origin and can be widely found in both psychological as well as philosophical writing, dating as far back as Plato (Evans and Frankish, 2009, p. 2). The sheer amount of dualistic theories has not made the search for a definition of intuition an easy one. Rather than searching for the *truth value* of intuition (i.e., “what intuition really is”), specifically investigating the different underlying processes (Glöckner and Witteman, 2010) as well as the characteristics of its operation (Ferguson et al., 2014) has been suggested as a more fruitful endeavor. Existing functional characterizations of intuitive processes differ somewhat from each other, arguably because the domains in which intuition operates are various and thus its characteristics tend to vary. One converging working definition has emerged over the years, however. This builds the foundation of the present work. Therein, intuition is proposed to rely on a (tacit) knowledge base which is acquired throughout one’s lifetime. It elicits the colloquially known “gut feeling.” That is, intuitive judgment relies on some type of metacognitive experience, such as a feeling of rightness or processing fluency (Thompson and Morsanyi, 2012; Proust, 2015), which lead the decision maker to her judgment or choice. The reasons for her judgment, however, remain elusive to the decision-maker. That is to say that intuition operates without the decision maker being conscious of the internal processes that are leading her (judgment) behavior (Hogarth, 2001; Gigerenzer, 2007; Plessner et al., 2008). The aim of the present work is to contribute to the functional characterization of intuition by tracing the cognitive processing characteristics of intuitive judgments in a face perception task using eye movement analysis.

Intuition has also been characterized as utilizing a global processing style and often related to processing the “gestalt” of an object rather than focusing on details. Dijkstra et al. (2014, 2012), for example, have demonstrated that the effects of decision mode (intuitive versus deliberate) on judgment are mediated by processing style. Their results suggest that similar mechanisms underlie intuition and global processing. Similarly, several recent studies have suggested that people may in some cases use a global or holistic strategy to process the information present in faces rather than relying on detailed features (e.g., Chuk et al., 2014). In the context of intuition, the term ‘holistic’ refers to the formation of an overall impression akin to the formation of a ‘gestalt’ (Wenger and Townsend, 2001; Dijkstra et al., 2012) based on rapidly gleaned and integrated information. This shares similarities with older definitions of holistic face processing as “recognizing the face as a perceptual whole” (Tanaka and Farah, 1993; see Maurer et al., 2002 for a review). Several highly cited works furthermore characterize intuition as a “holistically associative” process (Hogarth, 2001; Dane and Pratt, 2007; Hodgkinson et al., 2008; Gore and Sadler-Smith, 2011). Thereby the authors intend that the intuitive process integrates unstructured parts of stimulus information into a coherent percept. This percept then leads to action tendencies, such as making a decision or judgment, based on the integrated information. In the case of face perception, the notion of an internal ‘face space’ (Valentine et al., 2016) might represent the proverbial ‘database’ against which the holistically sampled percept is matched rapidly and non-consciously.

Eye movement strategies themselves have also been shown to rely on either global or local information sampling for the perception of faces and observers can flexibly adapt these strategies (Miellet et al., 2011, 2013). This speaks for the importance of individual differences in face perception strategies, though culture has repeatedly been shown to modulate these strategies strongly (see Miyamoto et al., 2006; Nisbett and Masuda, 2007; Kelly et al., 2010, as well as Peterson and Eckstein, 2013). Cognitive processing styles or modes, such as intuition, range among such individual factors, which purportedly influence eye movement patterns during the perception of human faces. In fact, several investigations have revealed two distinct viewing strategies between participants, even though all participants were instructed equally. In an eye-tracking task requiring participants to judge the femininity of presented stimulus faces (Armann and Bülthoff, 2009) two sub-groups emerged without differential instructions: one group of participants who preferentially fixated on the eye region, and a second group who fixated on the center of the face more often and for longer. Together with participants’ verbal reports, the authors interpreted the group that showed longer and more centralized fixations as a separate, more holistic strategy. Interestingly, these participants themselves reported performing the task “intuitively” and as trying to gain an “overall impression.”

In keeping with the characterization of intuition mentioned above, a set of fewer but longer fixations falling around the central axis of a face are in line with gaining an overall impression (or gestalt) by way of a global/holistic overview of the face.

Chuk et al. (2014) arrive at a functional distinction between a holistic and an analytical strategy of face perception as well, using a face recognition task in Asian participants. The authors modeled participants’ eye movement patterns using hidden Markov models (HMMs; Chuk et al., 2014). Participants were asked to recognize previously learned faces in a set of new ones, while their eye movements were being recorded. A HMM assumes that the system which is being modeled is a process with hidden states. The association of observable data and prior hidden states are summarized using probability distributions, which represent the likelihood of a hidden state generating the observed data. By clustering the HMMs, whereby each hidden state represents a different ROI of a face and the directly observable data represent fixation locations, participant eye movements could be classified into either a holistic or an analytic pattern. A more condensed fixation pattern on the center of the face was interpreted as ‘holistic pattern’ (as opposed to an analytical pattern, consisting of fixation areas on both eyes and the mouth). Furthermore, the participants classified as analytic by the HMMs showed a higher number of fixations and longer reaction times.

These findings are in line with literature on eye movement patterns of experts. Therein, longer fixation times are interpreted as a function of processing efficiency. Several investigations of experts in various areas such as chess, art and goal-keeping have found longer and fewer fixations in experts than in novices (Savelsbergh et al., 2002; Charness et al., 2005). The authors interpret this finding as experts extracting more information around the point of fixation (thus the longer fixation time) and therefore needing less fixations overall. Conversely, novices, who – due to lack of skill – will extract less information per fixation (shorter fixations) and thus need more fixations overall to complete the task (Reingold et al., 2001). Notably, expertise (especially domain-specific) is linked to intuitive processing, though intuition and expertise are not identical (cp. Dane and Pratt, 2007; Moxley et al., 2012).

To our knowledge, the question whether intuitively judging facial expressions maps onto a global/holistic viewing strategy has not been directly probed. We therefore set out to study eye movement patterns during intuitive face judgments, since this methodology is known to “provide an objective insight into the information entering the visual system and into cognitive processes involved” (Armann and Bülthoff, 2009). To this end, we differentially instructed two groups of participants: an “intuitive group,” whom we instructed to judge the authenticity of facial expressions relying on their “gut feeling” and “answering spontaneously.” As well as a “deliberate group,” whom we instructed to judge the authenticity of (the same) facial expressions after careful thought and focusing especially on the eye and mouth region (see Materials and Methods for explicit instructions). The present work relies on the design that has been successfully used to investigate intuitive processing using fMRI methodology (Mega et al., 2015). Furthermore, the direct instruction of decision mode in a between-subject design follows the methodological recommendations of leading experts in the field (Horstmann et al., 2009b). We presented 171 happy and fearful faces (342 total stimuli of various ages and genders) and asked participants to judge how authentic they perceived the facial expression to be. We hypothesized that, if intuitive judgments of faces rely on a global/holistic processing style, the intuitive condition should elicit fewer fixations in total and the attention map of the intuitive group should conform to a global/holistic pattern of perception. That is, the fixation pattern should be narrower/condensed and cluster around the center of the stimulus (face), rather than conforming to a featural processing strategy, i.e., fixating predominantly the eyes and the mouth region. Conversely, we would expect the intuitive group to show the same pattern as the deliberate one (this being the classical pattern of face processing found in Caucasian individuals), if the intuitive processing of facial expressions does not rely on a holistic perception strategy.

## Materials and Methods

### Participants and Instruction

Forty-three healthy, right-handed volunteers were included in this study (32 females). The age range was 19–35 years (mean [*M*] age: 25.87). Seven participants chose to not disclose gender and age. Participants were compensated with 10 Euros per hour for their participation. Handedness was tested using the Edinburgh Handedness Survey. Eighteen participants dropped-out, of which 15 were due to technical difficulties during scanning, or because of data loss due to non-completion of the entire experimental session. Three participants were excluded from analysis because post session questioning revealed a non-adherence to instruction. By non-adherence, we refer to participants who in the debriefing or in the post-session questionnaire mentioned being unable to follow the given instruction until the end, or explicitly stated using a strategy that was opposed to the given instruction. For example, one person who was given the instruction of the deliberate group reported relying on their gut feeling and first impression to make the authenticity judgment. This resulted in 25 participants in total (13 in the intuitive, 12 in the deliberate group). This study was carried out in accordance with the recommendations of the local ethics committee of the University of Tuebingen with written informed consent from all subjects. All subjects gave written informed consent in accordance with the Declaration of Helsinki. The protocol was approved by the same committee. Data was handled anonymously. All participants were native German speakers, reported no history of neuropsychiatric disorders, and were not currently taking psychoactive medications. Participants were pseudo-randomly assigned to two conditions: In the *intuitive* group, participants received the following instruction:

“Your task is to judge the emotional expression you will see with regard to its authenticity (realness)… Previous studies have shown that people are good at judging the authenticity (realness) of a smiling or fearful expression if they follow their initial feeling, that is, answer spontaneously and without thinking for too long. We therefore ask you to make your judgment quickly, and most importantly, to follow your first feeling, thus deciding ‘based on your gut.’”

In contrast, the instruction for the *deliberate* group was as follows:

“Your task is to judge the emotional expression you will see regarding its authenticity (realness)…Previous studies have shown that people are good at judging the authenticity (realness) of a smiling or fearful expression if they analyze and study the expression well, that is, think about their answer. Therefore, before you respond, study the expression thoroughly—within the given time! Most importantly, pay attention to the matching of the facial muscles in the eye and mouth regions”.

This instruction of strategy relies on a design that has been successfully used to investigate intuitive processing using fMRI methodology (Mega et al., 2015) and is proposed as standard in the field (Horstmann et al., 2009b). Similar wording has also been used in other tasks probing face judgments (Rule et al., 2009). A feature-based face processing strategy has reliably been shown for individuals of the age range and ethnicity of our participants. By asking participants to focus on the eye- and mouth region, we therefore simply explicitly instructed them to focus on the features we expected that these types of individuals are known to focus on. The deliberate group is therefore a kind of control condition.

### Apparatus

Eye movements were recorded at a sampling rate of 220 Hz with the Arrington ViewPoint Eyetracker, using a chin and forehead rest. Only the dominant eye was tracked (monocular tracking). The experiment was implemented in Matlab (2012b The MathWorks, Natick, MA, United States), using the Psychophysics Toolbox (PTB-3). Calibrations of eye fixations were conducted at the beginning of the experiment using a nine-point fixation procedure using ViewPoint software. Calibrations were then validated with the ViewPoint software and repeated when necessary until the optimal calibration criterion was reached.

### Task Outline

**FIGURE 1:**
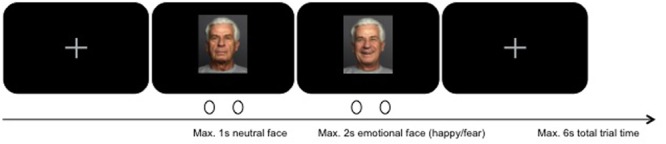
Overview of trial design using exemplary stimulus from the FACES database kindly provided by Ebner et al. (2010).

The experiment consisted of 340 stimuli, showing either a happy or a fearful facial expression. Stimuli were taken from the FACE database established by Ebner et al. (2010) and presented at 600 × 750 pixels image size on black background. Participants viewed the stimuli from 51 cm distance, on a monitor with a screen resolution of 1920 × 1080 pixels. Participants were tasked with indicating whether they perceived the facial expression to be authentic or not (yes/no response assignment was balanced across participants). The 170 happy and 170 fearful facial expressions were presented, wherein gender and age group of the lay actors in the stimulus pictures (“young” [*M* = 24.2 years, *SD* = 3.4; range 19–31], “middle-aged” [*M* = 49.0 years, *SD* = 3.9; range 39–55], and “57 years and older” [*M* = 73.2 years, *SD* = 2.8; range 69–80] as classified by Ebner et al., 2010) were balanced across conditions. Happy and fearful facial expressions were presented in blocks of ten, resulting in 34 blocks across the entire experiment. All trials lasted for 6 s: after a short fixation (variable duration), the neutral facial expression of the respective lay actor was shown for 1 s, followed by the presentation of the emotional facial expression, which was either shown for a maximum of 2 s, or for as long as participants took to make their choice (response-dependent abortion; see **Figure 1**). For the remaining time of the trial, a fixation cross was presented. Finally, participants were debriefed and thanked.

### Data Processing and Analysis

Raw eye tracking data was processed by automatically detecting blinks, as well as dropped frames, and removing the resulting artifacts. A running average was used to interpolate data between the start and end points of the blink artifacts. Fixation events were classified using the I-DT algorithm as introduced by Salvucci and Goldberg (2000) with the modifications proposed by Blignaut (2009). Based on recommendations in the literature, the thresholds applied were 100 ms (min. time) and 0,8° visual angle (dispersion). Dependent variables were number of fixations and fixation duration (throughout the stimulus space), as well as the data-driven, statistically established attention map (i.e., viewing pattern) of both groups separately and in comparison. Global eye-tracking measures (number of fixations and fixation duration) were calculated using IBM SPSS Version 22 (IBM Corporation and Others, 2013). The statistical fixation maps were computed with the *i*Map toolbox (version 3, Caldara and Miellet, 2011), running on Matlab 2014b (The MathWorks, Natick, MA, United States). *i*Map establishes significance using a robust statistical approach correcting for multiple comparisons in the fixation map space. A one-tailed Pixel test (Chauvin et al., 2005) was applied for the group fixation maps (*p* < 1,0) and a two-tailed Pixel test (*p* < 0.05) on the differential fixation maps. Finally, for each condition average *Z*-score values were extracted for each observer individually, within the regions showing significance in the differential fixation maps.

### Manipulation Check

To assure that participants in the two groups did rely on the instructed strategy (intuitive/deliberate), we compared the response latencies for the two conditions. Indeed, participants in the intuitive group were significantly faster in judging the authenticity of facial expressions than participants in the deliberate condition: *F*(1,21): 8,050; *p* = 0,010.

## Results

### Global Eye-Tracking Measures

#### Number of Fixations

A repeated measures ANOVA testing the number of fixations on the entire stimulus (including only those pixels wherein at least eight fixation events occurred) revealed a significant difference between the intuitive and the deliberate group: *F*(1,21) = 5.520, *p* = 0.028. The mean number of fixations per group on the stimulus was 3.596 (intuitive) and 5.135 (deliberate). Thus, the intuitive group showed fewer overall fixations on the face stimuli than the deliberate group.

#### Fixation Duration

The analysis of fixation durations between the two groups revealed a tendency for longer fixations in the intuitive conditions, albeit this difference did not reach statistical significance: *F*(1,21) = 3.553, *p* = 0.073. The mean fixation duration per group on the stimulus was 0.183 s (deliberate) and 0.211 s (intuitive). Neither the test for the effect of expression (i.e., happy or fearful), nor the interaction effect between expression and group revealed any significant differences in the fixation count or duration.

### Pixel-Wise Statistical Analysis (*i*Map3)

**FIGURE 2:**
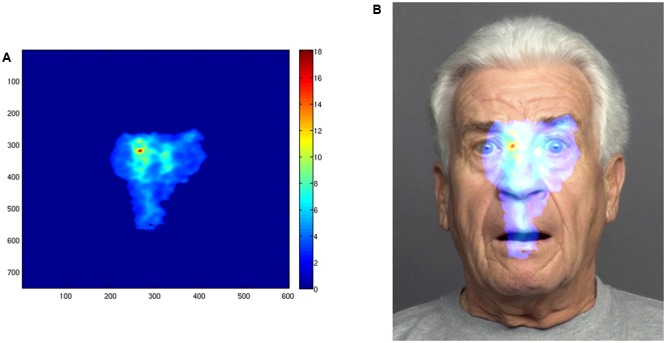
Pixel-wise statistical map showing the number of fixations in the stimulus space of the intuitive group as revealed by the *i*Map3 analysis. **(A)** The statistical pattern of distribution of fixations. The colors of the map correspond to fixation counts on that particular area (see color scale on the right). **(B)** The same pattern mapped onto an example stimulus. A one-tailed Pixel test (Chauvin et al., 2005) was applied for the group fixation map (*p* < 1,0). Finally, for each condition average *Z*-score values were extracted for each observer individually, within the regions showing significance in the differential fixation maps.

**FIGURE 3:**
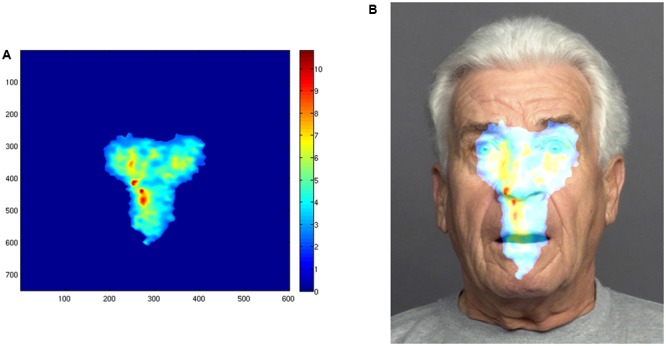
Pixel-wise statistical map showing the number of fixations in the stimulus space of the deliberate group as revealed by the *i*Map3 analysis. **(A)** The statistical pattern of distribution of fixations. The colors of the map correspond to fixation counts on that particular area (see color scale on the right). **(B)** The same pattern mapped onto an example stimulus. A one-tailed Pixel test (Chauvin et al., 2005) was applied for the group fixation maps (*p* < 1,0). Finally, for each condition average *Z*-score values were extracted for each observer individually, within the regions showing significance in the differential fixation maps.

We used the power of *i*Map3 as statistical mapping method for fixation data to represent and compare the distribution of the number and of the duration of the fixations on the face stimuli. We collapsed the fixation data from all face stimuli into one category, to compare and contrast overall viewing patterns, resulting in two fixation maps (fixation duration and number of fixations) for each individual. We then grouped the individual fixation maps by instruction to compute *Z*-scores on a pixel-by-pixel basis, resulting in *Z*-score statistical maps (**Figures 2, 3**) allowing for direct comparison of the two conditions. This data-driven method allows for direct comparisons of the differential viewing patterns (also referred to as attention map) between the two instruction groups, thus enabling us to go beyond the AOI approach.

#### Attention Map

For the intuitive group, the viewing pattern as revealed by the *i*Map analysis is narrow and centralized in the stimulus space (**Figures 2A,B**). In contrast, the attention map of the deliberate group shows several areas of significant attention clustered around the eyes, mouth, and nose regions (**Figure 3**).

Since fixation durations have been shown to be highly idiosyncratic and judgment strategy itself already is a highly individualized marker, we focus here on the more robust number of fixations to compare the two judgment conditions. The viewing patterns as revealed by fixation duration are analogous, however.

### Additional Measures

**FIGURE 4:**
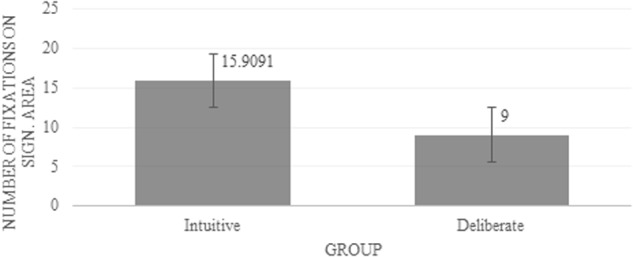
Average number of fixations on the significant area. Error bars indicate standard error.

**FIGURE 5:**
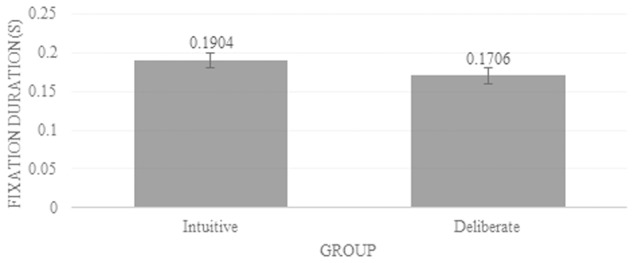
Average duration of fixations on the significant area. Error bars indicate standard error.

For each condition, we extracted the average descriptive values (i.e., number of fixation [**Figure 4**] and fixation duration [**Figure 5**]) for each observer individually, within the regions showing significance in the differential fixation maps.

## Discussion

We set out to further characterize intuition by directly investigating intuitive processing in a motivationally salient task. Intuitive processing is often related to processing the “gestalt” of an object rather than focusing on details (e.g., Shapiro and Spence, 1997; Epstein and Pacini, 1999). While a *local* processing style is related to a focus on details and concrete features, when in a *global* processing style, people make sense of a stimulus by integrating it into superordinate knowledge structures (Dijkstra et al., 2014). In this vein, several recent studies have suggested that people may in some cases use a global/holistic strategy to process the information present in faces rather than relying on detailed features. To our knowledge, the question whether using one’s intuition to judge facial expressions maps onto a global viewing strategy has not been directly probed. To this end, we set out to study the eye movement patterns of two differently instructed groups of participants: an “intuitive group,” whom we instructed to judge the authenticity of facial expressions relying on their “gut feeling” and “answering spontaneously.” As well as a “deliberate group,” whom we instructed to judge the authenticity of (the same) facial expressions after careful thought and focusing especially on the eye and mouth region.

The viewing pattern of the intuitive group is distinct from the deliberate one, confirming the elicitation of a difference in strategy by direct instruction (see Horstmann et al., 2009b for recommendations on using direct instructions when investigating intuition). In addition to confirming our manipulation, the fixation pattern conforms to theory-based expectations, which suggest the use of a global information search strategy in intuitive processing. The following arguments shall clarify this conclusion in detail.

### Centralized Attention Map in Intuitive Condition

The attention map revealed by the data-driven *i*Map analysis provides validation for the finding of global/holistic processing in the intuitive condition. The attention map of the intuitive group is centralized within the face-stimulus space, with the highest number of fixations (i.e., the area of greatest attention) localized around the area of the face midline (between the eyebrows, nose, and mouth). The deliberate condition, on the other hand, conforms to the instructed viewing strategy, landing on both the eyes and the mouth region and generally more spread out across the stimulus-space. This pattern constitutes the average pattern of face perception, reliably found for young Caucasian individuals viewing static face stimuli in eye-tracking studies (e.g., Sæther et al., 2009). Furthermore, several face perception studies, which did not directly instruct differential viewing modes, nevertheless found separable viewing patterns interpreted to be differential viewing strategies (cp. Armann and Bülthoff, 2009; Chuk et al., 2014).

#### Reliability of **D**ata by the **U**se of **D**ata-**D**riven **A**pproach with *i*Map3

Areas (or regions) of interest in eye tracking studies are often defined manually by the investigator and thereby what is termed as the “nose” in one study might well correspond to the area defined as “left eye” in another. For example, Barton et al. (2006) defined the mouth region as irregularly shaped ROI around the mouth, whereas (2005) included part of the cheek in their definition of the “mouth” ROI. Thus, eye movements of participants to the cheek would be defined as landing on the “mouth” in one study, but not in the other [see the Eye Data Quality (EDQ) Standardisation Project^1^ of the COGAIN Network of Excellence for an attempt at unifying method-wide standards of measure]. To avoid this confusion and lack of generalizability, we used a data-driven approach based on pixel-wise statistical comparisons with multiple comparison correction (*i*Map, Version 3, Caldara and Miellet, 2011). This approach allows for robust direct comparisons of the differential scanning patterns between conditions.

The analysis revealed areas of significant difference between the two conditions in the number of fixations, located in the center of the stimulus space. In other words, the center of the face was fixated significantly more often in the intuitive condition, than in the deliberate one. The distribution of fixations in the deliberate condition was more distinctly localized on the eye, nose, and mouth region of the stimulus faces. Thus, this condition shows less fixations landing on the center on the face than the intuitive one. We take these findings as further evidence in support of the hypothesis that intuitively judging faces relies on global/holistic face processing.

### Significantly Fewer, but Relatively Longer Fixations in the Intuitive Condition

The finding of fewer fixations for the intuitive as opposed to the deliberate condition is in line with previous findings investigating intuitive and deliberate judgment processes using eye-tracking, albeit in a lexical task (Horstmann et al., 2009a). Therein, participants were presented with city pairs and asked to decide which of the two cities have more inhabitants. Since the cities were given arbitrary names (e.g., city A), participants made their judgments based on concurrently presented probabilistic cues, such as the presence or absence of an airport. The authors found significantly less fixations in the intuitive than the deliberate group, as well as a higher percentage of inspected information for the deliberate group. However, it is imperative to not interpret the number of fixations and fixation duration in isolation of the fixation locations (viewing pattern). The (average) three fixations of the intuitive group could have also landed only on the eye region (cp. Armann and Bülthoff, 2009), or the eyes and mouth. If it was simply the difference in judgment speed that underlies the viewing differences between the two groups, that pattern would be expected. Instead, the few fixations required for the intuitive group to make their judgments fell in a centralized location of the stimulus faces, in accordance with our theoretical predictions. Intuitive face judgments seem to rely on “focusing on the forest rather than the trees,” or in this case, forming a holistic gestalt-like impression of the face rather than focusing on specific local featural cues (such as eyes, mouth or nose). Making few (but relatively long) fixations in a centralized location of the face can give a general impression of the facial expression.

### Intuitive Face Judgment Uses Global Viewing Pattern

When investigating face perception mechanisms of Western-Caucasians, as well as participants of a ‘young’ age group (i.e., around the mean age of the participants in this study, i.e., 25.87), the viewing pattern typically found is a feature-based one. That is, young Westerners usually seem to rely more on local information in the face (mostly the eye and mouth region) especially when compared with the viewing pattern of Asian participants (cp. Kelly et al., 2010 and Miellet et al., 2013 for an overview of the effects of culture on eye movement strategies). This may seem contrary to the earlier argument, that face perception happens intuitively. However, when we say that face perception is normally done intuitively, what we refer to is the perception of faces “in the wild.” Conversely, we believe that a laboratory context may very well induce a more deliberate mode of processing, thereby resulting in the average finding of a featural face processing strategy in Western-Caucasian individuals. In our opinion, it is important to keep in mind that most face perception studies do not ask for or directly investigate cognitive strategy, in the sense of intuitive or deliberate processing. We therefore often cannot know which processing mode or strategy individuals were engaging in these instances. Some evidence that participants alter their strategy, if they spontaneously decide to intuitively perform face judgments, can be found (e.g., as mentioned above for the study by Armann and Bülthoff, 2009). This may hint at the other participants being in a more deliberate processing mode. Though we can only speculate about this possibility, since it was not the focus of the investigation.

Since the present study was conducted solely with participants of a Western-Caucasian cultural background, one could expect a local, feature-based processing strategy (focusing on eye- and mouth region) for both instruction groups. Therefore, we take the differential markers for holistic/global processing revealed in the intuitive condition (fewer overall fixations, centrally located in the face) to be a specific function of the instructed judgment condition. Seeing as global viewing strategies of faces have been demonstrated reliably as an East Asian viewing pattern, the present investigation raises the question whether East Asians might rely more on their intuition to view faces than people of a different cultural background. This question, however, is beyond the purview of the present study and will need to be investigated more in the future.

### Questions for Future Research

From research on eye movement patterns in reading, a quite well documented effect is the extrafoveal sampling of information in the stimulus. Recently, Miellet and Caldara (2012) and Miellet et al. (2013) showed that the sampling of extrafoveal information also plays a role in face recognition. Therefore, finding a centralized fixation pattern may point toward the sampling of the other cues in the face extrafoveally. Since we did not directly investigate this matter, we can only speculate on the involvement of extrafoveal sampling in the present study. We believe, however, that extracting extrafoveal information does not speak against intuitive processing being a distinguishable viewing pattern. Rather, sampling of information that is not directly fixated conforms to the characterization of intuition, describing it as a process whereby information is sampled but does not reach consciousness (Bowers et al., 1990; Horr et al., 2014; Mega et al., 2015). Further investigations are needed to shed light on the role of extrafoveal information sampling for intuitive face judgments.

Differential viewing strategies are also discussed as a function of task demands and individual differences. Within the community studying intuition, individual differences have long been recognized as a key factor. Since the characteristics demarcating intuition (automatic processes relying on a tacit knowledge base that reaches consciousness through some form of metacognitive experience (e.g., fluency), thereby leading the decision-maker to her judgment) are heavily based on internal representations, it is not surprising that individual difference effects should play a role. Furthermore, Miellet et al. (2013) argue for task-induced differences, a logic which we very much agree with (see Introduction).

Since we believe the centralized location of the area of significant viewing difference between the two conditions to be a function of global processing, we do not make inferences as to the role of this specific facial region for the differences in face judgments. We would like to refrain from speculation about the role of the fixated regions, especially because no reliable community-standard of measurement and location yet exists for eye tracking studies (as opposed to fMRI studies, for example, which make use of anatomical atlases such as the Talairach Atlas [Talairach and Tournoux, 1988]). However, the gaze contingent expanding spotlight method has recently been introduced as a means to assess the visual processing of peripheral versus central retinal inputs (Miellet et al., 2013). We hope that in the future this method may provide insight into understanding not only which locations in the face are fixated but also which of the fixated information reaches consciousness. A further interesting avenue would be to investigate, whether the intuitive viewing pattern of faces can be replicated using different task types and participant groups. If a global viewing strategy can reliably be established as intuitive across task-types and modalities, one more characteristic of intuitive processing will have been found.

### Limitations of This Study

The small sample size, due to the large amount of drop-out, is a limitation of this study. While the results of the present work should therefore be interpreted conservatively, they are in line with theoretical predictions for intuitive face judgments and present a further case for the global/holistic nature of intuitive processes.

The wording of the instructions for the deliberate group is another limitation of the present study, since it manipulates the viewing location directly. However, the rationale for the specific instruction to focus on the eye and mouth region was two-fold. Firstly, deliberate processing has been associated with a sequential information search strategy (Betsch, 2008). By instructing participants to focus on specific features of the face, we intended to induce this strategy. For the same reason this instruction was used in our previous fMRI study (Mega et al., 2015), which relied on the same study design as the present work. However, we remain confident that the results of the present study are reliable and relevant to the field of intuition. The foremost reason for this being that the eye movements of the intuitive group differed significantly from those of the deliberate one, not only in scan path but also in the number of fixations. These differences fall into the realm as hypothesized based on current literature in the field. Moreover, the scan pattern we instructed does not differ from eye movements typically found for face perception in a Western-European context. Nevertheless, future studies, which do not rely on the use of our previously investigated design, should refrain from using an instruction which directly mentions the scan pattern. Especially if, contrary to the present work, the focus of the future study is on the characterization of the deliberate process.

### What Does This Mean for the Study of Intuitive Processing?

To our knowledge, these results constitute one of very few studies that directly investigate intuitive judgment processes in the context of a socially relevant task. Intuitive processes rely on a (tacit) knowledge base acquired throughout one’s lifetime. Being surrounded by faces and the need to quickly glean meaning from facial categories and expressions all our lives, it is unsurprising that having a global impression of a facial expression might well be enough to elicit a “gut feeling” of the message we interpret a face to be sending. Only those having undergone explicit training in subtle expression detection or micro expression detection (Ekman, 2006) consciously can retrieve the knowledge about which muscle interplay leads to what expression (though there seem to be some naturals, see O’Sullivan and Ekman, 2005). Nevertheless, as humans we move through social spaces and have natural conversations with each other, relying on our intuition to interpret others’ facial expressions for successful social interactions.

## Conclusion

In the present work, we have shown that participants who are asked to listen to their gut feeling and spontaneously judge whether they perceive the facial expression they are presented with as authentic, reveal markers of global/holistic processing. These are a pattern of attention localized in the center of the face, as well as a significantly lower number of fixations as compared to the deliberate condition. This, to our knowledge, constitutes one of the first studies linking intuition and holistic processing in a socially, and thereby motivationally salient task. Of course, further studies using diverse ways of operationalizing intuition as well as different task-types are necessary to validate our findings. Insofar as intuition and deliberation can be considered two different processing styles for the information within the faces of others, it seems quite plausible to postulate that intuitive and deliberative processing strategies will differ in the pattern of attention on a given face. The present study provides further evidence that intuitive processes rely on holistic perception, in an understudied and real world domain of intuition research. Additionally, our work adds to a growing body of literature demonstrating the usefulness of eye-tracking technology for judgment and decision-making research in general (e.g., Russo, 2011) and intuition in particular (Horstmann et al., 2009a; Thompson, 2013).

## Author Contributions

Both authors equally contributed to the conception of the ideas and design of the experiment. LM implemented the experiment. LM and KV together analyzed the data, discussed the results, and contributed to writing the manuscript.

## Conflict of Interest Statement

The authors declare that the research was conducted in the absence of any commercial or financial relationships that could be construed as a potential conflict of interest. The reviewer EA and handling Editor declared their shared affiliation, and the handling Editor states that the process nevertheless met the standards of a fair and objective review.

## Footnotes

1 http://www.cogain.org/info/eye-data-quality

## References

[B1] Ambady N. (2010). The perils of pondering: intuition and thin slice judgments. *Psychol. Inq.* 21 271–278. 10.1080/1047840X.2010.524882

[B2] Armann R. Bülthoff I. (2009). Gaze behavior in face comparison: the roles of sex, task, and symmetry. *Atten. Percept. Psychophys.* 71 1107–1126. 10.3758/APP.71.5.1107 19525541

[B3] Barton J. J. S. Radcliffe N. Cherkasova M. V. Edelman J. Intriligator J. M. (2006). Information processing during face recognition: the effects of familiarity, inversion, and morphing on scanning fixations. *Perception* 35 1089–1105. 10.1068/p5547 17076068

[B4] Betsch T. (2008). “The nature of intuition and its neglect in research on judgment and decision making,” in *Intuition in Judgment and Decision Making* eds Plessner C. Betsch H. Betsch T. (Mahwah, NJ: Lawrence Erlbaum) 3–22.

[B5] Blignaut P. (2009). Fixation identification?: the optimum threshold for a dispersion algorithm. *Atten. Percept. Psychophys.* 71 881–895. 10.3758/APP 19429966

[B6] Bowers K. S. Regehr G. Balthazard C. Parker K. (1990). Intuition in the context of discovery. *Cogn. Psychol.* 22 72–110. 10.1016/0010-0285(90)90004-N

[B7] Caldara R. Miellet S. (2011). *i*Map: a novel method for statistical fixation mapping of eye movement data. *Behav. Res. Methods* 43 864–878. 10.3758/s13428-011-0092-x 21512875

[B8] Charness N. Tuffiash M. Krampe R. Reingold E. Vasyukova E. (2005). The role of deliberate practice in chess expertise. *Appl. Cogn. Psychol.* 19 151–165. 10.1002/acp.1106

[B9] Chauvin A. Worsley K. J. Schyns P. G. Arguin M. Gosselin F. (2005). Accurate statistical tests for smooth classification images. *J. Vis.* 5 659–667. 10.1167/5.9.1 16356076

[B10] Chuk T. Chan A. B. Hsiao J. H. (2014). Understanding eye movements in face recognition using hidden markov models. *J. Vis.* 14:8 10.1167/14.11.8 25228627

[B11] Dane E. Pratt M. G. (2007). Exploring intuition and its role in managerial decision making. *Acad. Manag. Rev.* 32 33–54. 10.5465/AMR.2007.23463682

[B12] Dijkstra K. A. van der Pligt J. van Kleef G. A. (2014). Effects of processing style on responsiveness to affective stimuli and processing fluency. *Cogn. Emot.* 28 959–970. 10.1080/02699931.2013.865597 24341779

[B13] Dijkstra K. A. van der Pligt J. van Kleef G. A. Kerstholt J. H. (2012). Deliberation versus intuition: global versus local processing in judgment and choice. *J. Exp. Soc. Psychol.* 48 1156–1161. 10.1016/j.jesp.2012.05.001

[B14] Ebner N. C. Riediger M. Lindenberger U. (2010). FACES–a database of facial expressions in young, middle-aged, and older women and men: development and validation. *Behav. Res. Methods* 42 351–362. 10.3758/BRM.42.1.351 20160315

[B15] Ekman P. (2006). Darwin, deception, and facial expression. *Ann. N. Y. Acad. Sci.* 1000 205–221. 10.1196/annals.1280.010 14766633

[B16] Epstein S. Pacini R. (1999). “Some basic issues regarding dual-process theories from the perspective of cognitive-experiential self-theory,” in *Dual-Process Theories in Social Psychology* eds Chaiken S. Trope Y. (New York: Guilford Press) 462–482.

[B17] Evans J. S. B. T. (2008). Dual-processing accounts of reasoning, judgment, and social cognition. *Annu. Rev. Psychol.* 59 255–278. 10.1146/annurev.psych.59.103006.093629 18154502

[B18] Evans J. S. B. T. Frankish K. (eds) (2009). *In Two Minds: Dual Processes and Beyond.* Oxford: Oxford University Press.

[B19] Ferguson M. J. Mann T. C. Wojnowicz M. (2014). “Rethinking duality: criticisms and ways forward,” in *Dual-Process Theories of the Social Mind* eds Sherman J. Gawronski B. Trope Y. (New York, NY: The Guildford Press) 578–594.

[B20] Gigerenzer G. (2007). *Gut Feelings: The Intelligence of the Unconscious.* New York City, NY: Viking Press.

[B21] Glöckner A. Witteman C. (eds) (2010). “Foundations for tracing intuitions. Models, findings, categorizations,” in *Foundations for Tracing Intuition: Challenges and Methods* (New York, NY: Psychology Press) 1–23.

[B22] Gore J. Sadler-Smith E. (2011). Unpacking intuition: a process and outcome framework. *Rev. Gen. Psychol.* 44 304–316. 10.1037/a0025069

[B23] Hodgkinson G. P. Langan-Fox J. Sadler-Smith E. (2008). Intuition: a fundamental bridging construct in the behavioural sciences. *Br. J. Psychol.* 99 1–27. 10.1348/000712607X216666 17559716

[B24] Hogarth R. M. (2001). *Educating Intuition.* Chicago, IL: University of Chicago Press.

[B25] Horr N. K. Braun C. Volz K. G. (2014). Feeling before knowing why: the role of the orbitofrontal cortex in intuitive judgments-an MEG study. *Cogn. Affect. Behav. Neurosci.* 14 1271–1285. 10.3758/s13415-014-0286-7 24789812 PMC4218982

[B26] Horstmann N. Ahlgrimm A. Glöckner A. (2009a). How distinct are intuition and deliberation?: an eye tracking analysis of instruction induced decision modes. *Judgm. Decis. Mak.* 4 335–354. 10.2139/ssrn.1393729

[B27] Horstmann N. Hausmann D. Ryf S. (2009b). “Methods for inducing intuitive and deliberate processing modes,” in *Foundations for Tracing Intuition: Challenges and Methods* eds Glöckner A. Witteman C. (Abingdon: Taylor & Francis) 219–237.

[B28] Kelly D. J. Miellet S. Caldara R. (2010). Culture shapes eye movements for visually homogeneous objects. *Front. Psychol.* 1:6 10.3389/fpsyg.2010.00006 PMC3153738 21833189

[B29] Maurer D. Le Grand R. Mondloch C. J. (2002). The many faces of configural processing. *Trends Cogn. Sci.* 6 255–260. 10.1016/S1364-6613(02)01903-4 12039607

[B30] Mega L. F. Gigerenzer G. Volz K. G. (2015). Do intuitive and deliberate judgments rely on two distinct neural systems? A case study in face processing. *Front. Hum. Neurosci.* 9:456 10.3389/fnhum.2015.00456 PMC4548224 26379523

[B31] Miellet S. Caldara R. (2012). When East meets West: gaze-contingent Blindspots abolish cultural diversity in eye movements for faces. *J. Eye Mov. Res.* 5 1–12. 10.1167/10.7.703

[B54] Miellet S. Caldara R. Schyns P. G. (2011). Local Jekyll and global hyde: the dual identity of face identification. *Psychol. Sci.* 22 1518–1526. 10.1177/0956797611424290 22075238

[B32] Miellet S. Vizioli L. He L. Zhou X. Caldara R. (2013). Mapping face recognition information use across cultures. *Front. Psychol.* 4:34 10.3389/fpsyg.2013.00034 PMC3576804 23430143

[B33] Miyamoto Y. Nisbett R. E. Masuda T. (2006). Culture and the physical environment. Holistic versus analytic perceptual affordances. *Psychol. Sci.* 17 113–119. 10.1111/j.1467-9280.2006.01673.x 16466418

[B34] Moxley J. H. Ericsson A. K. Charness N. Krampe R. T. (2012). The role of intuition and deliberative thinking in experts’ superior tactical decision-making. *Cognition* 124 72–78. 10.1016/j.cognition.2012.03.005 22541584

[B35] Nisbett R. E. Masuda T. (2007). Culture and point of view. *Intellectica* 46 153–172.

[B36] O’Sullivan M. Ekman P. (2005). “The wizards of deception detection,” in *Detecting Deception in Forensic Contexts* eds Granhag P. A. Stromwell L. (Cambridge: Cambridge University Press) 269–286.

[B37] Peterson M. F. Eckstein M. P. (2013). Individual differences in eye movements during face identification reflect observer-specific optimal points of fixation. *Psychol. Sci.* 24 1216–1225. 10.1177/0956797612471684 23740552 PMC6590077

[B38] Plessner H. Betsch T. Betsch C. (eds) (2008). *Intuition in Judgment and Decision Making.* Mahwah, NJ: Lawrence Erlbaum Assoc Inc.

[B39] Proust J. (2015). “The representational structure of feelings,” in *Open MIND* eds Metzinger T. Windt J. M. (Frankfurt: MIND Group) 10.15502/9783958570047

[B40] Reingold E. M. Charness N. Schultetus R. S. Stampe D. M. (2001). Perceptual automaticity in expert chess players: parallel encoding of chess relations. *Psychon. Bull. Rev.* 8 504–510. 11700901 10.3758/bf03196185

[B41] Rule N. O. Ambady N. Hallett K. C. (2009). Female sexual orientation is perceived accurately, rapidly, and automatically from the face and its features. *J. Exp. Soc. Psychol.* 45 1245–1251. 10.1016/j.jesp.2009.07.010

[B42] Russo J. E. (2011). “Eye fixations as a process trace,” in *A Handbook of Process Tracing Methods for Decision Research* eds Schulte-Mecklenbeck M. Kuehberger A. Ranyard R. (Hove: Psychology Press) 43–64.

[B43] Sæther L. Van Belle W. Laeng B. Brennen T. Øvervoll M. (2009). Anchoring gaze when categorizing faces’ sex: evidence from eye-tracking data. *Vis. Res.* 49 2870–2880. 10.1016/j.visres.2009.09.001 19733582

[B44] Salvucci D. D. Goldberg J. H. (2000). “Identifying fixations and saccades in eye-tracking protocols,” in *ETRA ’00 Proceedings of the 2000 Symposium on Eye Tracking Research and Applications* Palm Beach Gardens, FL 71–78. 10.1145/355017.355028

[B45] Savelsbergh G. J. Williams A. M. Van Der Kamp J. Ward P. (2002). Visual search, anticipation and expertise in soccer goalkeepers. *J. Sports Sci.* 20 279–287. 10.1080/026404102317284826 11999482

[B46] Shapiro S. Spence M. (1997). Managerial intuition: a conceptual and operational framework. *Bus. Horiz.* 40 63–68. 10.1016/S0007-6813(97)90027-6

[B47] Talairach J. Tournoux P. (1988). *Co-planar Stereotaxic Atlas of the Human Brain.* New York: Thieme.

[B48] Tanaka J. W. Farah M. J. (1993). Parts and wholes in face recognition. *Q. J. Exp. Psychol.* 46 225–245. 10.1080/14640749308401045 8316637

[B49] Thompson V. Morsanyi K. (2012). Analytic thinking: do you feel like it? *Mind Soc.* 11 93–105. 10.1007/s11299-012-0100-6

[B50] Thompson V. A. (2013). Why it matters: the implications of autonomous processes for dual process theories–commentary on Evans & Stanovich (2013). *Perspect. Psychol. Sci.* 8 253–256. 10.1177/1745691613483476 26172968

[B51] Valentine T. Lewis M. B. Hills P. J. (2016). Face-space: a unifying concept in face-recognition research. *Q. J. Exp. Psychol.* 69 1996–2019. 10.1080/17470218.2014.990392 25427883

[B52] Wenger M. J. Townsend J. T. (2001). “Faces as gestalt stimuli: process characteristics,” in *Computational, Geometric, and Process Perspectives on Facial Cognition: Contexts and Challenges. Scientific Psychology Series* eds Wenger M. J. Townsend J. T. (Hove: Psychology Press) 229–284.

[B53] Witteman C. Van den Bercken J. Claes L. Godoy A. (2009). Assessing rational and intuitive thinking styles. *Eur. J. Psychol. Assess.* 25 39–47. 10.1027/1015-5759.25.1.39

